# Low adherence with antihypertensives in actual practice: the association with social participation – a multilevel analysis

**DOI:** 10.1186/1471-2458-5-17

**Published:** 2005-02-18

**Authors:** Kristina Johnell, Lennart Råstam, Thor Lithman, Jan Sundquist, Juan Merlo

**Affiliations:** 1 Centre for Family Medicine, Karolinska Institutet, Huddinge, Sweden; 2 Department of Community Medicine, Malmö University Hospital, Lund University, Malmö, Sweden; 3 Regional Office, Skåne County Council, Lund, Sweden

## Abstract

**Background:**

Low adherence is a key factor in explaining impaired effectiveness and efficiency in the pharmacological treatment of hypertension. However, little is known about which factors determine low adherence in actual practice.

The purpose of this study is to examine whether low social participation is associated with low adherence with antihypertensive medication, and if this association is modified by the municipality of residence.

**Methods:**

1288 users of antihypertensive medication were identified from The Health Survey in Scania 2000, Sweden. The outcome was low adherence with antihypertensives during the last two weeks. Multilevel logistic regression with participants at the first level and municipalities at the second level was used for analyses of the data.

**Results:**

Low social participation was associated with low adherence with antihypertensives during the last two weeks (OR = 2.05, 95% CI: 1.05–3.99), independently of low educational level. However, after additional adjustment for poor self-rated health and poor psychological health, the association between low social participation and low adherence with antihypertensives during the last two weeks remained but was not conclusive (OR = 1.80, 95% CI: 0.90–3.61). Furthermore, the association between low social participation and low adherence with antihypertensives during the last two weeks varied among municipalities in Scania (i.e., cross-level interaction).

**Conclusion:**

Low social participation seems to be associated with low adherence with antihypertensives during the last two weeks, and this association may be modified by the municipality of residence. Future studies aimed at investigating health-related behaviours in general and low adherence with medication in particular might benefit if they consider area of residence.

## Background

The effectiveness [1,2] and efficiency [3] of antihypertensives may be questioned, as adherence with antihypertensives may be as low as 50% [1,4]. The efficacy of antihypertensives has been evaluated in randomised clinical trials (RCTs). The RCTs are often of short duration, the study population is usually carefully selected and patients with co-morbidity or advanced ages are often excluded from these trials [5-8]. Furthermore, even though drop-outs and lost to follow up occur in RCTs, adherence with medication treatment is often actively supported. In actual practice, however, many patients, who would be excluded from RCTs, receive medication [2] for a long time and may not be as adherent with medication as those included in RCTs [5].

Low adherence is one important cause of uncontrolled hypertension [9,10]. Yet, low adherence is sometimes unrecognised [11] and is often interpreted as treatment resistance [10,12,13]. However, little is known about which factors determine low adherence in actual practice [14,15]. The purpose of this study is to examine whether low social participation is associated with low adherence with antihypertensives during the last two weeks, and if this association is modified by the municipality of residence.

Social participation is an important concept for understanding the influence of social factors on individual health and behaviour, and can be viewed as a feature of individual social networks [16]. Good social networks have been suggested to influence health behaviours, possibly through information exchange and establishment of health-related group norms [17]. Accordingly, a high level of social participation may facilitate adherence [16] and this association could be modified by the area of residence [18,19].

Multilevel analysis handles both the micro-scale of people and the macro-scale of context simultaneously within one model [20]. This analytic approach has been suggested as an interesting tool in pharmacoepidemiology [21].

The first aim of this study is to examine whether low social participation is associated with low adherence with antihypertensives during the last two weeks, independently of low educational level and health status (i.e., poor self-rated health and poor psychological health). The second aim is to analyse whether the hypothesised association between low social participation and low adherence with antihypertensives during the last two weeks varies between municipalities in Scania.

## Methods

### Participants

The Health Survey in Scania 2000 (HSS-2000) was a postal self-administered questionnaire sent out to a random sample of 23 437 individuals born from 1919 to 1981 living in Scania. The purpose of the HSS-2000 was to obtain information about health conditions and different types of health hazards among the inhabitants of Scania [22]. The province of Scania in southern Sweden has a population of about 1.2 million inhabitants and is divided into 33 municipalities. In total, 59% participated, of which 98% had complete information about medication use. The present study focused on those 9.6% who indicated use of antihypertensives during the last year and who had complete information about social participation (n = 1288).

The Ethical Committee at the Medical Faculty of Lund University approved the study proposal of The HSS-2000, and all of the participants received written information about the survey.

### Outcome variable

Use of antihypertensives was based on an affirmative answer to the question "Have you during the last year used medicine, which was bought at the pharmacy...?" and indicating "Medication for high blood pressure"

*Low adherence with antihypertensives during the last two weeks *(dichotomised) was based on the question "Have you used (this) medicine during the last year, but not during the last 2 weeks?" Those participants who answered yes were considered to have low adherence.

### Explanatory variables

*Age *was categorised in five groups: <35 (reference), 35–44, 45–54, 55–64 and ≥ 65 years.

*Low social participation *(dichotomised) was assessed after the respondent stated involvement in three or fewer activities (lowest quartile) of 13 formal or informal activities (study circle/course at work place, other study circle/course, union meeting, meeting of other organisations, theatre/cinema, arts exhibition, church, sports event, letter to editor of a newspaper/journal, demonstration, night club/entertainment, large gathering of relatives, private party), which the respondent might have participated in during the previous 12 months [23].

*Low educational level *(dichotomised) was defined as having nine years of education or less.

*Poor self-rated health *(dichotomised) was defined as a value of ≤ 3 on an ordinal self-rated health scale ranging from 1 ("Very bad") to 7 ("Very good") [24].

*Poor psychological health *(dichotomised) was determined by giving three or more affirmative answers to the 12 items composing the Standardised General Health Questionnaire (GHQ-12) [25].

### Statistical analysis

Because of the hierarchy in the data, with individuals nested in municipalities, we used multilevel logistic regression [26] with individuals at the first level and municipalities at the second level. The area of residence might affect a person's social participation [27], and, consequently, it may be possible that the influence of low social participation on low adherence with antihypertensives during the last two weeks may vary between municipalities. Therefore, we let the slopes of the association between low social participation and low adherence vary at the municipality level. This random slopes analysis gives information about whether the association between low social participation and low adherence is different in different municipalities.

The first model *i *was created to study the influence of low social participation on low adherence with antihypertensives during the last two weeks, adjusting for age and sex. The second model *ii *was extended to also include low educational level, because low educational level could be a confounder in the association between low social participation and low adherence with antihypertensives during the last two weeks. The third model *iii *additionally contained poor self-rated health and poor psychological health. Impaired health may affect both low social participation and low adherence with antihypertensives.

#### Fixed effects

The results are shown as odds ratios (OR) with 95% confidence intervals (CI)

#### Random effects

We calculated the second level variance (variation between municipalities) regarding prevalence of low adherence with antihypertensives during the last two weeks (i.e., the intercepts in the multilevel regression), and the second level variance regarding the association between low social participation and low adherence with antihypertensives during the last two weeks (i.e., the slope variance in the multilevel regression). We also calculated the covariance between intercept and slope residuals. The covariance gives information about whether the association between low social participation and low adherence with antihypertensives during the last two weeks depends on the prevalence of low adherence in the different municipalities (i.e., cross-level interaction).

Parameters were estimated using the Restricted Iterative Generalized Least Squares (RIGLS) and penalised quasilikelihood (PQL). Extra-binomial variation was explored systematically in all models and we found no evidence for under- or over-dispersion. The MLwiN, Version 1.1 software package [28] was used for the analyses.

## Results

Low adherence with antihypertensives during the last two weeks was found among 11% (145/1 288) of the participants and 49% (635/1 288) were classified as having low social participation. The participants mean age was 63 years. Those participants classified as having low social participation more often reported low adherence with antihypertensives during the last two weeks, low educational level, poor self-rated health and poor psychological health than those who were not classified as having low social participation (Table 1).

**Table 1:** Characteristics of the participants (n = 1288) according to individual low social participation.

	Low social participation		
	Yes (n = 635) No. (%)	No (n = 653) No. (%)	Total (n= 1288) No. (%)

			
Age (mean years)	65	60	63
Women	340 (54)	346 (53)	686 (53)
			
Low adherence with antihypertensives	96 (15)	49 (8)	145 (11)
Low educational level	417 (70)	284 (45)	701 (57)
			
Poor self-rated health	112 (19)	64 (10)	176 (14)
Poor psychological health	124 (21)	95 (15)	219 (18)

### Fixed effects

Participants with low social participation had on average a more than twofold higher probability of reporting low adherence with antihypertensives during the last two weeks than those who did not have low social participation (OR = 2.28, 95% CI: 1.16–4.49) (Table 2). This association between low social participation and low adherence with antihypertensives during the last two weeks persisted after adjusting for low educational level (OR = 2.05, 95% CI: 1.05–3.99). However, after additional adjustment for poor self-rated health and poor psychological health, the association between low social participation and low adherence with antihypertensives during the last two weeks was not conclusive using a 95% confidence interval (OR = 1.80, 95% CI: 0.90–3.61).

**Table 2:** Municipality variance and age adjusted odds ratios (95% confidence intervals) of low adherence with antihypertensives during the last two weeks in relation to sex, low social participation, low educational level, poor self-rated health and poor psychological health.

	Model i		Model ii		Model iii	
	**OR**	**95% CI**	**OR**	**95% CI**	**OR**	**95% CI**

						
Women vs. men	1.21	(0.82–1.78)	1.15	(0.78–1.69)	1.17	(0.78–1.77)
Low social participation (yes vs. no)	2.28	(1.16–4.49)	2.05	(1.05–3.99)	1.80	(0.90–3.61)
Low educational level (yes vs. no)			1.87	(1.18–2.96)	1.76	(1.09–2.84)
Poor self-rated health (yes vs. no)					1.45	(0.83–2.54)
Poor psychological health (yes vs. no)					1.54	(0.92–2.59)
						

	**Variance**	**SE**	**Variance**	**SE**	**Variance**	**SE**

Municipality variance in low adherence with antihypertensives (intercept variance)	0.801	(0.461)	0.776	(0.450)	0.793	(0.467)
Municipality variance of the association between low social participation and low adherence with antihypertensives (slope variance)	1.812	(0.889)	1.720	(0.857)	1.799	(0.916)
Municipality covariance between intercepts and slopes	-1.163	(0.609)	-1.116	(0.591)	-1.175	(0.625)

### Random effects

**Figure 1:**
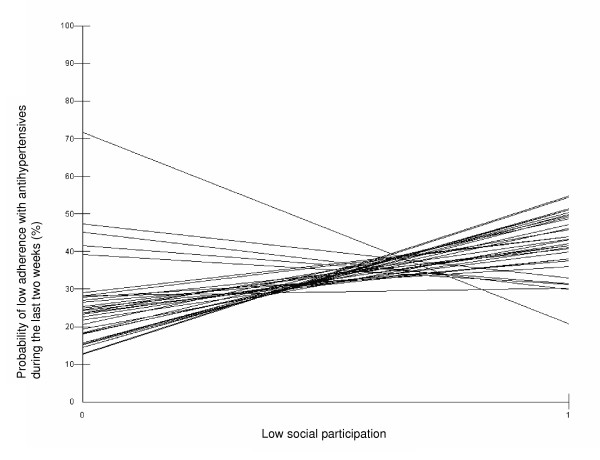
Slope variance in the association between low social participation and low adherence with antihypertensives during the last two weeks among 33 municipalities in Scania, Sweden.

We found a variance between the municipalities in both low adherence with antihypertensives during the last two weeks (intercept variance) and in the association between low social participation and low adherence with antihypertensives during the last two weeks (slope variance) (Table 2 and Figure 1). The negative covariance between intercepts and slopes (Table 2) suggested that the associations between low social participation and low adherence with antihypertensives during the last two weeks (slopes) in the 33 municipalities depended on different prevalence of low adherence with antihypertensives during the last two weeks in the different municipalities. The association between low social participation and low adherence with antihypertensives during the last two weeks (slope) was weaker in municipalities with higher prevalence than in municipalities with lower prevalence of low adherence with antihypertensives during the last two weeks (i.e., cross-level interaction).

## Discussion

### Main findings

Our results suggest that low social participation is associated with low adherence with antihypertensives during the last two weeks, independently of low educational level. In other words, the association between low social participation and low adherence withstood adjustment for socio-economic position (expressed by educational level) in our analyses. Social participation might therefore be considered as a real construct, and not only a proxy for socio-economic position. However, the association between low social participation and low adherence with antihypertensives during the last two weeks was weakened after additional adjustment for poor self-rated health and poor psychological health. Furthermore, the association between low social participation and low adherence with antihypertensives during the last two weeks may vary between municipalities in Scania.

The weakening of the association between low social participation and low adherence with antihypertensives during the last two weeks after we adjusted for poor self-rated health and poor psychological health may be an expression for confounding. Impaired health may negatively affect both social participation and adherence with antihypertensives. On the other hand, the observed reduction of the association may instead be telling us that physical and mental health are in the pathway between low social participation and low adherence with antihypertensives during the last two weeks [29]. Social participation may be considered as an early factor in the causal pathway that determines individual health-related behaviour, such as low adherence with medication. Social networks, which are connected to social participation, may promote shared norms around health behaviours, as treatment adherence, which could explain the pathway between social networks and impaired health [16].

Our present finding that the association between low social participation and low adherence with antihypertensives during the last two weeks may vary between municipalities in Scania gives empirical support to the existence of cross-level interactions (i.e., between municipality and individual) associated with health-related behaviours, such as low adherence with medication. These behaviours may be a result of the interaction between a person and his or her area of residence [18]. In a previous analysis, we observed that both individual and neighbourhood social participation are associated with individual impaired health and with use of hormone replacement therapy in women [18].

The present study shows that multilevel regression analysis can be used for investigation of geographical disparities in health and health-related behaviour (e.g, adherence with medication), without analysing any specific area characteristic [30]. In multilevel analysis, area effects can be investigated by measures of variance and by examining how area boundaries modify individual level associations [31].

### Limitations of the study

The rather low participation rate (59%) may increase the risk of selection bias and reduce the ability to generalise the results and compare them to other populations. Nevertheless, the participation rate for participants aged 51–80 was about 65% and the participants using antihypertensives had a mean age of 63 years.

The low adherence question we used was stated in a non-threatening manner, which might facilitate for participants to give an honest response and not underreport low adherence [32]. Self-reported adherence has been reported to correlate with clinical measures of disease activity and control [4]. Moreover, self-report offers a convenient and non-invasive estimate of adherence behaviour. Nevertheless, the procedure of measuring adherence is controversial. Self-report can be subject to self-presentational and recall biases. People may overestimate their adherence and their memory may be inaccurate [32]. We might have reduced memory bias in this study by restricting the recall time to two weeks. However, the prevalence of current low adherence (11%) in this study is lower than low adherence reported in a longer period of time, which may be as high as 50% [1,4]. Therefore, our results may underestimate the association between social participation and low adherence with antihypertensives. It is possible that some participants with high adherence in the last two weeks had low adherence in other periods of the year. If this kind of misclassification would be more frequent among participants with low social participation, there would be differential misclassification, and the association between low social participation and adherence with antihypertensives could be underestimated. Non-differential misclassification would also underestimate the association between low social participation and low adherence with antihypertensives. Other ways of measuring adherence may be more appropriate, such as Morisky's four-item scale [33], which will be used in the Health Survey for Scania 2004.

People with low social participation and low adherence with antihypertensives during the last two weeks may have been less inclined to respond to the HSS-2000 questionnaire. This possible selection bias could lead to an underestimation of the association between low social participation and low adherence with antihypertensives during the last two weeks.

## Conclusion

Our results suggest that low social participation is associated with low adherence with antihypertensives during the last two weeks, independently of low educational level. In addition, the association between low social participation and low adherence with antihypertensives during the last two weeks seems to vary between the municipalities in Scania, which gives empirical support to the existence of cross-level interactions (i.e., between municipality and individual) associated with health-related behaviours, such as low adherence with medication. We have recently showed that factors related to the area of residence influence the individual blood pressure level, especially in people using antihypertensive medication [34], which is in concordance with the results of this present study.

Future studies aimed at investigating health-related behaviours in general and low adherence with medication in particular might benefit if they consider that area of residence may modify associations between individual variables.

## Competing interests

The author(s) declare that they have no competing interests.

## Authors' contributions

JM and KJ developed the original idea, participated in the design of the study, performed the statistical analyses and drafted the manuscript. LR and JS participated in the design of the study and revised the manuscript. TL participated in the design of the study, helped to collect the data and revised the manuscript. All authors read and approved the final manuscript.

## Pre-publication history

The pre-publication history for this paper can be accessed here:

http://www.biomedcentral.com/1471-2458/5/17/prepub
